# Local and global control adjustments to stimulus-based task conflict in task switching

**DOI:** 10.1177/17470218231200442

**Published:** 2023-09-28

**Authors:** Luca Moretti, Iring Koch, Stefanie Schuch

**Affiliations:** Institute of Psychology, RWTH Aachen University, Aachen, Germany

**Keywords:** Task conflict, dual mode of control, cognitive control, task switching

## Abstract

A prominent feature of cognitive control is that its deployment is regulated depending on the environmental circumstances. Control upregulation has been widely documented in response-conflict paradigms where congruency effects are reduced both following incongruent trials, and in blocks where incongruent trials are the majority. In two pre-registered task-switching experiments, we investigated whether similar flexible mechanisms are also available when dealing with stimulus-based task conflict. Building up on previous Stroop studies, task conflict was measured as the difference in performance between bivalent congruent and univalent trials, which we name the “valency effect.” If cognitive control is upregulated analogously to what observed with response conflict, valency effects should be reduced following bivalent trials and in majority-bivalent blocks. Furthermore, as cognitive control upregulation has been proposed to be task specific, we assessed whether switching to a new task eliminates the expected modulations of task. The results broadly matched our predictions. First, we observed a reduction of the valency effect following bivalent trials similar to the well-known congruency sequence effect, demonstrating similar patterns of flexible control adjustment to task and response conflict. This valency sequence effect was limited to task repetitions, indicating that local control adjustments are task specific. Furthermore, task conflict was reduced in majority-bivalent blocks, similar to the proportion-congruency effect. This finding extends previous Stroop studies suggesting that control is recruited proactively when dealing with stimulus-based task. The proportion valency effect was limited to task-switch trials, leaving open the question on the precise mechanisms behind sustained control adjustments.

Cognitive control has been investigated in the past decades using a variety of experimental tasks. Although paradigms eliciting conflict between responses (i.e., the Simon, the Stroop and the flanker paradigms) are arguably the most employed to this purpose, conflict between tasks is also worth investigating (Schuch et al., 2019). In previous studies, our group has compared response conflict and task conflict with regard to their causes (Moretti et al., 2023b) and their consequences on subsequent behaviour (Moretti et al., 2021, 2023a), finding notable dissociations between the two. In this work, we focus on the mechanisms employed to solve task conflict, again drawing a parallel with the better-investigated response conflict.

In particular, we asked whether cognitive control can be upregulated when dealing with stimulus-based task conflict depending on factors known to modulate response conflict, such as conflict history and conflict likelihood. To this aim, we used manipulations conceptually similar to those employed in response-conflict tasks for eliciting cognitive control upregulations. Furthermore, in line with current debates on the generalisability of cognitive control (Braem et al., 2014), we asked whether control adjustments to task conflict would be specific or general in nature. Using a task-switching paradigm, we assessed whether solving conflict in one task is advantageous also in the context of the other task (i.e., task-general control), or whether control upregulations are only effective in task-repetition trials (i.e., task-specific control).

In what follows, we start by briefly reviewing the literature on response conflict, highlighting the hallmarks of control upregulation in response-conflict tasks. We then review similar findings regarding task conflict in the Stroop literature, suggesting that cognitive control is upregulated proactively when stimulus-based task conflict is frequent. Finally, we relate both strands of research to the task-switching paradigm, presenting the results of two pre-registered experiments using this task.

## Response conflict and control upregulation

Modern theories of cognitive control revolve around the concept of cognitive conflict (Botvinick et al., 2001), which may be defined as the simultaneous activation of two or more cognitive representations when only one can be selected (Norman & Shallice, 1986). Oftentimes, conflict results from the co-activation of goal-directed behaviour, which is in line with one person’s intentions, and automatic behaviour, which is triggered by environmental stimuli. For example, a person accustomed to driving on the right-hand side would experience conflict when driving in the United Kingdom. In this situation, the person’s habits (e.g., using the left lane to overtake) contrast with the action schemas required to reach the current goal (e.g., using the right lane for overtaking when in the United Kingdom). Therefore, our tourist experiences conflict, or, more specifically, *response conflict* as two responses are in competition to achieve his intention (i.e., overtaking).

In the lab, response conflict has long been studied using paradigms that are designed to elicit competition between automatic and goal-directed responses, such as the Stroop (1935), the Simon (1990), and the flanker task (Eriksen & Eriksen, 1974). For example, in the Stroop task, participants are required to name the colour of the letters of a colour word. Response conflict is present in incongruent trials, where the word meaning diverges from the colour (e.g., the word RED presented in blue), and is absent in congruent trials, where the same response is activated by both stimulus dimensions (e.g., RED presented in red). Because of response conflict, performance is usually worse in incongruent compared with congruent trials. Therefore, the resulting congruency effect is commonly interpreted as a measure of the time taken by control mechanisms to solve such response conflict (MacLeod, 1991; however, see Rey-Mermet et al., 2018).

A prominent feature of the cognitive control mechanisms dealing with response conflict is that their deployment changes flexibly according to environmental demands. Depending on the likelihood of conflict occurrence, cognitive control is recruited in either a sustained or a transient fashion (Braver, 2012; Braver et al., 2007). In the first case, control is said to be *proactive* because it is recruited prior to conflict occurrence. Proactive control is exerted by biasing attention to the task-relevant feature in a sustained fashion, resulting in reduced stimulus-based interference. For example, participants may use the inter-trial interval to prepare for a colour judgement in the Stroop task. If, instead, control is recruited only after conflict detection, it is said to be *reactive*. In this case, no active preparation process is assumed to occur prior of stimulus onset and attentional control would depend exclusively on whether conflict is encountered. Reactive control can thus be thought as a just-in-time mechanism that is advantageous in case conflict is relatively unlikely, or if error commission is not so damaging for the subject (Chiew & Braver, 2014; Locke & Braver, 2008).

Given the central role of conflict likelihood in eliciting proactive control, investigations of this phenomenon have largely focused on manipulating the proportion of congruent trials blockwise (Bugg & Chanani, 2011; Bugg & Gonthier, 2020; Gonthier et al., 2016; see Bugg & Crump, 2012, for a review). Congruency effects are present in blocks where congruent trials are the majority (typically ca. 70%–80%), whereas they are reduced or absent when most trials are incongruent (Bugg, 2014; Gonthier et al., 2016; Logan & Zbrodoff, 1979). This proportion of congruency effect (herein PCE) indicates that when response conflict is likely (i.e., in majority-incongruent blocks), participants proactively prepare for it. This causes performance to improve in incongruent trials, where interference is reduced, but to be worse in congruent trials, where the irrelevant dimension cues the correct response.

Similarly, cognitive control can be regulated also depending on immediate conflict history. When the previous trial is incongruent, congruency effects are again reduced compared with post-congruent trials (Gratton et al., 1992), a phenomenon known as the congruency sequence effect (herein CSE). Whether the CSE stems from proactive or reactive control recruitment is still a matter of debate. In their first report of the CSE, Gratton and colleagues (1992) attributed the effect to a bias in expectations about congruency in the following trial. Therefore, in its first interpretation, the CSE was attributed to proactive mechanisms (Correa et al., 2009; Schiltenwolf et al., 2023). However, more recent studies mostly tend to attribute the CSE to the lingering effects of reactive control upregulation in the previous trial (Duthoo et al., 2014; Duthoo & Notebaert, 2012; Egner et al., 2010; Scherbaum et al., 2012; Yang & Pourtois, 2022). Particularly relevant to this debate is the finding that CSE tends to decrease with increasing inter-trial interval (Duthoo et al., 2014; Egner et al., 2010; however, see Schiltenwolf et al., 2023), which indicates that the processes involved in generating this effect are transient rather than sustained. Whether the CSE is due to proactive or to reactive control, the clear difference in comparison to the PCE is that the former is due to trial-by-trial control upregulation, whereas the latter is due to a more sustained processing mode. We therefore refer to the CSE as arising from local control mechanisms and to the PCE from global control mechanisms.

## Task conflict vs. response conflict

The evidence reviewed so far indicates that response conflict is solved through control mechanisms whose recruitment is flexibly adjusted to the environmental circumstances. In recent years however, many contributions have emphasised the value of investigating cognitive control beyond the response level, by focusing on conflict between task representations (Schuch et al., 2019). A number of methods have been developed to this aim using formal modelling of behavioural data (Moretti et al., 2023a, 2023b; Steinhauser & Hübner, 2009), markers of neural activity (Desmet et al., 2011; Elchlepp et al., 2013), and measures of pupil dilation (Hershman et al., 2021; Hershman & Henik, 2019). The picture emerging from these studies points to both similarities and differences between task-level and response-level control. In a recent study from our group for example, only task conflict (but not response conflict) was found to be affected by the salience of the task-irrelevant stimulus dimension in task switching (Moretti et al., 2023b), indicating that the triggers of task and response conflict can be dissociated. Furthermore, failures in task control, but not in response control, have been shown to affect measures of task-set inhibition (Moretti et al., 2021, 2023a) and cognitive flexibility (Steinhauser & Hübner, 2006, 2008). Finally, the time-course of the control mechanisms dealing with the two types of conflict differs. While response conflict is quickly suppressed showing its effects in fast responses, task conflict is mainly observable in slow trials (Shahar & Meiran, 2015; Steinhauser & Hübner, 2009).

Given the reported dissociations between task and response conflict in terms of their causes, consequences, and control mechanisms, in this study, we ask whether also the employment of control strategies (i.e., sustained and reactive control) would differ, or would rather be similar. The Stroop literature may partially help us in addressing such question. In the Stroop task, task conflict can be dissociated from response conflict in congruent trials, where the irrelevant word dimension triggers the correct response, but the wrong task (i.e., reading; MacLeod & MacDonald, 2000; Monsell et al., 2001; for recent reviews, see Littman et al., 2019; Parris et al., 2022; however, see Parris et al., 2023). Therefore, comparing congruent and univalent trials should hold a valency effect such that (bivalent) congruent trials would result in slower responses than univalent trials. Surprisingly however, the opposite pattern is usually observed. To account for this finding, Goldfarb and Henik (2007) proposed that in the Stroop task, proactive control is exerted so strongly to leave no trace of task conflict on behaviour. To test this idea, the authors manipulated the proportion of univalent trials blockwise under the assumption that proactive control would be relaxed in majority-univalent blocks. In line with their expectations, the valency effect was indeed found in blocks with mostly univalent trials, but not in blocks where the majority of trials were congruent, suggesting that proactive control is indeed strongly used also to deal with task conflict. Since then, this finding has been replicated in a number of studies using the manual Stroop (Kalanthroff et al., 2013; Shichel & Tzelgov, 2018, Experiment 1), the vocal Stroop (Entel et al., 2015; Entel & Tzelgov, 2018; Kinoshita et al., 2018; Shichel & Tzelgov, 2018 Experiment 2), and the affordance task (Littman & Kalanthroff, 2021).

However, we wish to point out here that manipulating the proportion of univalent trials in a block creates a confound that is critical to the interpretation of the results. Although such blockwise manipulations are intended to elicit/relax sustained control, they also end up affecting the recruitment of control at the local level by changing the occurrence of some trial-by-trial sequences. For example, while introducing many univalent trials is supposed to relax the level of task-set control at the global level, this manipulation also increases the number of trials following a univalent trial. In other words, sequences of the kind univalent-univalent (uU) or univalent-bivalent (uB) will be present more often in blocks where most trials are univalent than in those where most trials are bivalent (for a similar argument for the PCE, see Torres-Quesada et al., 2014). If lack of task conflict in trial *N* − 1 led to a down-regulation of cognitive control, we would expect valency effects in trial *N* because of trial-by-trial adjustments in control rather than global mechanisms. At the same time, in majority-congruent blocks, we would expect the opposite pattern. In other words, if a valency sequence effect (VSE) existed, similar to the CSE, this could also account for the observed results, questioning the proactive nature of the control mechanisms dealing with task conflict.

Even though other studies have demonstrated the use of proactive control in the Stroop paradigm without manipulating the proportion of univalent trials in a block (Entel & Tzelgov, 2020; Kalanthroff et al., 2013, 2015, 2016; Parris, 2014), this is still an important issue to address, given that most Stroop research on stimulus-based task conflict has relied on this manipulation (cf. Parris et al., 2023). Therefore, the first aim of our study was to replicate the finding of higher task conflict in majority-univalent blocks while controlling for possible sequential effects.

## Task conflict and proactive control in task switching

Although the Stroop task has occasionally been used to investigate task, the task-switching paradigm is indubitably the main tool that researchers have employed to this purpose (for recent reviews, see Koch et al., 2018; Koch & Kiesel, 2022). In task switching, participants are required to alternate between simple classification tasks such as indicating the stimulus’ colour or the stimulus’ shape. The core finding is that performance is worse when the task switches compared with repetition trials, producing so-called switch costs.

Due to its multitasking nature, two sources of task conflict have been identified in this paradigm. The first is carryover interference from the previously relevant task. Once a task has been executed, its activation carries over to the next trial, thus causing interference in task switches (Allport et al., 1994). To avoid perseverative behaviours, some authors have proposed that participants actively prepare the next task before stimulus onset (Rogers & Monsell, 1995). In line with this idea, switch costs are reduced when participants are given more time to prepare (Meiran, 1996; Rogers & Monsell, 1995), suggesting that proactive control processes are recruited for dealing with carryover interference in task switches.

The second source of task conflict, akin to the Stroop task, is the use of bivalent stimuli, which causes stimulus-based task conflict (Allport & Wylie, 2000; Monsell et al., 2001). While carryover task interference is attenuated using proactive control, it is not clear whether this is also the case for stimulus-based task conflict. Only a few studies have investigated this issue so far. On one hand, Kalanthroff and Henik (2014) found that the valency effect got smaller with increasing cue to stimulus interval (CSI), suggesting that participants prepare for stimulus-based task conflict before stimulus onset, as soon as they process the cue indicating the upcoming task. On the other hand, the same interaction between valency and CSI did not replicate in a later study where valency was manipulated between subjects (Hirsch et al., 2016). Similar mixed results were obtained in two studies by Braverman and Meiran (2010, 2015), who took a different approach to measuring stimulus-based task conflict. In their studies, participants first performed a regular cued task-switching session with bivalent stimuli. In a second phase, univalent stimuli were used, and the cues previously associated with each task were now presented as distractor along with the target. Task congruency was then defined depending on whether such distractor was previously used to cue the alternative task (task incongruent), or whether it was used to cue the currently relevant task (task congruent). Although the task congruency effect was found to decrease with preparation time in one study (Braverman & Meiran, 2010), manipulating the proportion of task congruent trials had no impact on this metric (Braverman & Meiran, 2015).

To sum up, while carryover interference is known to be modulated by preparation, it is yet not clear whether participants can use proactive control to deal with stimulus-based task conflict in task switching. This stands in contrast with the Stroop literature, where proactive control is commonly thought to be used to this aim (Kalanthroff et al., 2018). On one hand, a discrepancy between these two paradigms is to be expected. While proactive control can be used to selectively suppress the irrelevant (or enhance the relevant) stimulus dimension in conflict tasks throughout a block, the same strategy cannot be used in task switching where the task unpredictably changes from one trial to the next. Therefore, global control effects observed in the Stroop task may not extend to task switching. On the other hand, manipulating the proportion of congruent trials did lead to significant PCEs in task-switching studies as well (Braverman & Meiran, 2015; Bugg & Braver, 2016; Schneider, 2015; Strobach et al., 2020; Wendt et al., 2013), suggesting that proactive control can also be used when the irrelevant task feature switches. In this study, we thus assessed whether global control effects are found in a task-switching paradigm when dealing with stimulus-based task conflict.

## Task specificity of response-conflict adaptations

An important question when characterising the control processes behind conflict adjustments is whether they reduce conflict caused by a specific source, or whether they can act to reduce conflict in a domain-general manner (Braem et al., 2014; Egner, 2017). This question is informative on some important properties of conflict monitoring. If control is exerted specifically on the source of conflict, the conflict monitoring mechanism(s) must first detect what the source of conflict is (Egner, 2008). If instead control is domain-general, the system can be agnostic as to what is causing conflict. In this second scenario, control could be achieved by a general increase in attention to the task-relevant features.

Previous studies have investigated this question by assessing whether congruency effects in one task (e.g., the Simon task) would be reduced following incongruent trials in another task (e.g., the flanker task). In other words, they investigated whether the CSE extends to a different task, which possibly generates conflict in a different fashion. The results generally indicated that the mechanisms recruited to deal with response conflict are specific rather than global (Akçay & Hazeltine, 2011; Egner, 2007; Funes et al., 2010; Kreutzfeldt et al., 2016; for a review, see Braem et al., 2014), although some exceptions have been reported (e.g., Kunde & Wühr, 2006). Importantly for the present context, the same findings were replicated using the classic task-switching paradigm (Kiesel et al., 2006; Schneider, 2015; Strobach et al., 2020; Wendt et al., 2013), where the irrelevant dimension of one task is the relevant dimension for the other. In the task-switching paradigm, preventing interference from one stimulus feature (e.g., colour) is not adaptive because in case of a task, the subsequent trial does not require to again suppress the same stimulus feature.

## The present study

In this study, we asked whether dealing with stimulus-based task conflict requires similar cognitive mechanisms as those employed with response conflict. In particular, we focused on the adaptive nature of cognitive control, with control being recruited more strongly following conflict trials (Gratton et al., 1992) and when conflict is likely to occur (Logan & Zbrodoff, 1979). The aim of this study was three-fold.

First, we aimed at providing evidence that reactive control mechanisms are also employed when dealing with stimulus-based task conflict (Experiment 1). Despite Stroop studies have mainly focused on proactive control and its global effects (e.g., Goldfarb & Henik, 2007; Kalanthroff et al., 2018), we believe that a just-in-time mechanism should also be present for dealing with task conflict suddenly evoked by the stimulus appearance. We predict, in line with the response-conflict literature, that the effects of such local control adjustments will linger on the next trial, thus producing a VSE.

Second, we intended to extend to task switching previous findings from the Stroop literature indicating that proactive control is effectively used when dealing with stimulus-based task conflict (Experiment 2), as indicated by differences between blocks of trials with mostly univalent versus mostly bivalent trials (proportion valency effect, PVE). We thus tested whether stimulus-based interference can also be dealt with proactively in a task-switching paradigm where the relevant and irrelevant stimulus dimensions may switch on every trial. Importantly, we did so by controlling for a possible confound between proportion of valency and *N* − 1 valency. Indeed, the VSE can also account for the findings of previous Stroop studies showing increased valency effects in low-conflict blocks (i.e., PVE), making it important to control for this possible confound.

Finally, we aimed at characterising the mechanisms behind both the PVE and the VSE in respect to their task-specific vs. task-general nature. In line with previous reports of the CSE being task-specific in task switching (e.g., Kiesel et al., 2006), we predict that the VSE will be limited to task-repetition trials. We aimed to explore whether this is also the case for the PVE, or whether the PVE occurs in both task-repetition and task-switch trials.

## Experiment 1

In Experiment 1, we investigated, for the first time, local control adjustments when confronting stimulus-based task conflict. We thus had participants to switch between a colour and a character task using an equal proportion of univalent and bivalent stimuli in each block. Drawing a parallel with the response-conflict literature, where response-conflict effects (i.e., the congruency effect) are reduced following response-conflict trials (i.e., following incongruent trials), we predicted to find a significant reduction of task-conflict effects (i.e., the valency effect) following task-conflict trials (i.e., bivalent trials). In other words, we expected to find a VSE. If control is upregulated following task conflict similarly as to what observed following response conflict, this effect should be confined to task-repetition trials.

Importantly, when assessing these questions, care must be taken to control for two important confounds. First, the CSE has been proposed to be vulnerable to episodic effects driven by the binding of perceptual and response features (Hommel et al., 2004; Mayr et al., 2003). To avoid this issue, we used a paradigm in which stimuli were never repeated within the same block. Second, as reviewed above, performance in congruent trials is typically slowed following incongruent trials (e.g., Egner et al., 2010). Therefore, analysing both post-congruent and post-incongruent trials may inflate the VSE. To solve this issue, one could consider avoid presenting incongruent trials altogether. Afterall, incongruent trials need to be discarded when assessing the valency effect anyway to isolate stimulus-based task conflict (present in both congruent and incongruent trials), from response conflict (present in incongruent trials). However, presenting exclusively congruent and univalent trials would likely eliminate the need of task selection altogether. This is because in univalent trials, only one task can be selected, whereas in congruent trials, participants may learn that attending to a specific task is irrelevant, as both tasks share the same response. Therefore, we chose to include incongruent stimuli in our experimental design, which we then discarded from analyses along with the subsequent trial.

### Methods

#### Participants

Sixty participants took part in the study. This number was chosen so to ensure that, even with the exclusion of a few participants (i.e., maximum eight), we would have a power of β = .80 for detecting a two-way interaction of size *d_z_* = 0.40.^1^ We chose this effect size not based on previous studies, as this procedure can suffer from publication bias, but rather as a standard effect in cognitive psychology (Brysbaert, 2019). The experiment was run and built online using Gorilla (Anwyl-Irvine et al., 2020).

#### Stimuli

The stimuli employed were strings of 10 coloured elements. Each element could either be the character “#” or “0.” Colours employed were orange and red. The participants’ task was either to indicate which character was most common in the array (i.e., #s or 0s, character task) or, alternatively, to indicate whether most elements were presented in red or orange colour (colour task). While bivalent stimuli had both a colour and a character dimension, and thus afforded both tasks, univalent elements only had one of them, being either composed of # and 0 characters printed in black or, alternatively, of Xs printed in orange and red (Kinoshita et al., 2017). Bivalent stimuli could either be congruent, if the task-relevant and the task-irrelevant dimension triggered the same response, or incongruent, if they did not.

Stimuli were built with the constraint that the ratio between elements of each feature would always be 6:4. For example, if the colour feature was present in the stimulus, six elements would be of one colour, and four would be of the opposite colour. We will refer to the level with the most elements within a feature as the majority level.

Univalent stimuli were built so that elements of neither level would ever be present more than twice in a row. For each combination of task (Colour, Character) and majority level (Feature A, Feature B), there were 43 stimuli that satisfied this criterion. Among them, 16 were chosen at random, resulting in a total of 64 univalent stimuli.

Bivalent stimuli were built applying the same rules to both stimulus’ features, with the additional constraint that minority-level elements could not be the first or the last element. There were 278 stimuli that satisfied this criterion for each combination of majority levels across features (Red-Hashtag, Red-Zero, Orange-Hashtag, Orange-Zero). Among them, 16 stimuli were chosen at random, and were employed during the experiment equally often in both tasks. This resulted in a total of 64 bivalent stimuli.

#### Trial procedure

**Figure 1.**
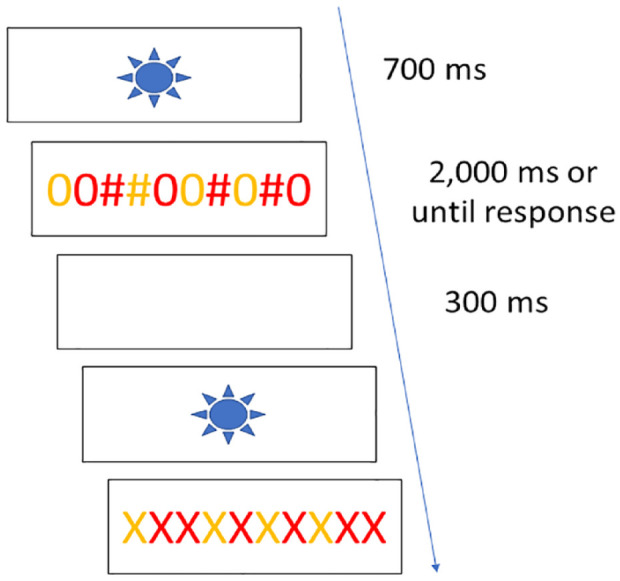
Experiment 1 and 2: trial procedure. The figure depicts the timing of events in each trial in both Experiments 1 and 2. First, the task cue was presented for 700 ms, followed by the stimulus which stayed on screen for 2,000 ms or until a response was selected. Finally, a blank screen appeared for 300 ms and a new trial began. The figure presents an example of a bivalent stimulus (in the first trial) and of a univalent stimulus for the colour task (in the second trial).

At the beginning of each trial, the task cue was presented at the centre of the screen for 700 ms (Figure 1). The drawing of a sun would indicate the colour task, whereas the drawing of a cloud was used for the magnitude task. After 700 ms elapsed, the cue was replaced by the stimulus, and participants were asked to respond within 2,000 ms: if they did respond within this limit, the screen turned blank for 300 ms and a new trial began. If they did not, the trial was considered a timeout, and again a new trial started after 300 ms. Responses were to be given by using the “A” and “L” keys of the keyboard. The stimulus–response mapping was counterbalanced, so that we used all four possible combinations.

The figure presents an example of a bivalent stimulus (in the first trial) and of a univalent stimulus for the colour task (in the second trial).

#### Experimental procedure

Participants could access the study by clicking on a link provided by the experimenter. After giving informed consent, the participants saw some instructions describing the task to be performed. The experiment began with two practice blocks of 16 trials each in which only one task was present. Following these pure blocks, the participants were familiarised with the task-switching procedure in another block of 64 trials in which both tasks were presented intermixed. During the whole practice phase, performance feedback was given after errors and timeout trials. The test phase consisted of nine blocks of 128 trials each. Performance feedback was no longer present. In all blocks, each stimulus was only presented once to avoid possible learning effects.

The sequence of task and stimuli was determined pseudorandomly so that in each block, there was an equal number of trials for each combination of task, sequence, majority level, and *N* − 1 valency separately for univalent and bivalent stimuli. Furthermore, for bivalent stimuli also the variable congruency was equally split for each of the abovementioned conditions. Finally, *N* − 1 congruence was kept balanced among all conditions including an *N* − 1 bivalent stimulus. A graphical representation of the number of trials in each condition is provided in Figure A1 of the Online Supplementary Material 1.

#### Data trimming and statistical design

Data trimming was implemented following the procedures specified in the pre-registration protocol: https://aspredicted.org/tx3jh.pdf. We started data trimming by excluding all participants whose error rate was higher than 33% (six participants) or producing more than 10% of suspiciously fast responses (0 participants), where a fast response was defined as Reaction times (RT) <200 ms. The remaining fast responses were excluded from analyses along with the subsequent trial. In addition, we excluded the first trial of each block, post-error trials, timeout trials, trials following a timeout, and, in the context of RT analyses, erroneous responses.

Most notably, in addition to such noisy trials, our design imposed to also remove incongruent and post-incongruent trials. The reason for excluding incongruent trials was that the valency effect should be assessed by contrasting univalent trials and bivalent congruent trials to be able to isolate task conflict from response conflict. However, *N* − 1 incongruent trials were excluded to avoid confounding the predicted VSE with the well-known CSE (Gratton et al., 1992).

Following data trimming, 41.6% of the trials were retained for the included participants. This low number is explained by the fact that by design, we forcefully had to exclude 56% of the trials (see Figure A1 in the Online Supplementary Material 1). For this reason, before running statistical analyses, we ensured that each participant had at least 15 trials in each cell of our design (as pre-registered). Data from one participant were excluded following this criterion.

Under a reviewer’s suggestion, we run linear mixed models (LMMs) for analysing RT data, and generalised linear mixed models (GLMMs) with logit link for analysing error data, thus deviating from our pre-registered analyses.^2^ For both analyses, we started by specifying a full model including the fixed effects Valency (bivalent, univalent), *N* − 1 Valency (bivalent, univalent), and Task Transition (repetition, switch). These effects were coded using contrast coding. The random component included by-participants random intercepts and slopes for the three-way interaction and all associated lower order effects. Following the specification of the full model, we used a backwards elimination procedure to select the most adequate random structure using the *buildmer* function of the buildmer package (Voeten, 2023). To ensure the robustness of our procedure, we repeated this step using three different elimination criteria: the likelihood-ratio test (as suggested by Matuschek et al., 2017), the Akaike information criterion (AIC), and the Bayesian information criterion (BIC). For the RT analyses, we inspected residuals’ normality through means of Q-Q plots. In case normality looked violated, we repeated the previous steps on log-transformed RTs. Although the inverse transformation (i.e., -1000/RT) is also sometimes used to this purpose (e.g., Schmidt, 2013), it has been argued that interaction terms are biased towards false negative results when using such transformations (Balota et al., 2013; see also Cohen-Shikora et al., 2019). After the model with the most adequate random structure was selected, its parameters were tested using the Kenward–Roger correction for degrees of freedom (Kenward & Roger, 2009), as advocated in a recent paper by Singmann and Kellen (2019). As this procedure is not available for GLMMs, in the error rate analyses, we used likelihood-ratio tests. Finally, we run planned contrasts with Holm correction for assessing the size of the valency effect in each of the four experimental conditions.

It is to be noticed that previous task-switching research has shown that univalent trials are indeed slower following bivalent trials, a phenomenon known as the *bivalency effect* (Grundy & Shedden, 2014; Meier et al., 2009; Rey-Mermet & Meier, 2012; Rey-Mermet et al., 2013; Woodward et al., 2003, 2008). These results have been interpreted as indicating that encountering infrequent bivalent stimuli would trigger a more cautious response style (Woodward et al., 2003, 2008, cf. Notebaert et al., 2009), or to reflect episodic context binding (Meier et al., 2009; Rey-Mermet & Meier, 2012). As slowing in univalent trials following a bivalent trial would also produce the expected Valency by *N* − 1 Valency interaction, without calling necessarily for control mechanisms, we specifically predicted that such an interaction should be driven by performance decrements in *N* − 1 univalent-bivalent trials rather than in *N* − 1 bivalent-univalent trials.

### Results

#### Reaction times

**Figure 2.**
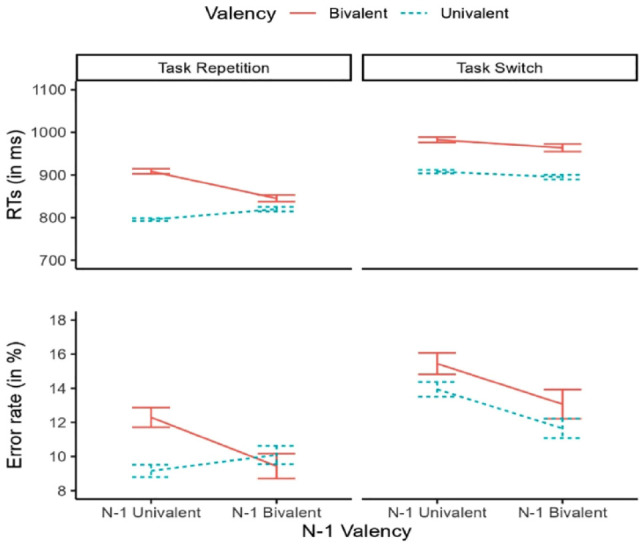
Experiment 1: descriptive statistics of the sample. Reaction times (upper panel) and error rates (lower panel) are plotted as a function of Valency (bivalent, univalent), *N* − 1 Valency (*N* − 1 bivalent, *N* − 1 univalent), and Task Transition (repetition, switch). Error bars represent standard error of the mean.

Descriptive statistics for both RTs and error rates can be found in Figure 2. The backward selection procedure held a random structure including by-subject random intercepts and correlated Task Transition and Valency slopes. The residuals of this model however were strongly skewed. We therefore log-transformed the RTs and proceeded again with the backward procedure. This step held the same results as with raw RTs, except that now the residuals were normally distributed. Our model can be described in Wilkinson notation as:

(1) logRT ~ Valency * *N* − 1 Valency * Task Transition + (Valency + Task Transition | PP)

This model revealed both a main effect of Task Transition, *F*(1, 58.0) = 208.43, *p* < .001, and a main effect of Valency, *F*(1, 54.7) = 140.70, *p* < .001, indicating the presence of switch costs and valency effects, respectively. The main effect of *N* − 1 Valency was also significant, *F*(1, 25,263) = 21.89, *p* < .001, due to slowing in post-univalent trials. Critical for our hypothesis, the two-way interaction between Valency and *N* − 1 Valency was also highly significant, *F*(1, 25,267) = 42.1, *p* < .001. This effect was further modulated by a three-way interaction involving all factors, *F*(1, 25.262) = 29.88, *p* < .001. To characterise this interaction, planned contrasts were run with appropriate Holm correction. In switch trials, valency effects were about the same size in post-bivalent (69 ms), *z* = 6.47, *p* < .001, and post-univalent *z* = 9.27, *p* < .001 (75 ms) trials. In repetition trials however, valency effects were greatly reduced for post-bivalent (26 ms), *z* = 3.14, *p* = .002, compared with post-univalent (113 ms), *z* = 15.64, *p* < .001 trials.

#### Error rates

The model selection procedure for the error rates converged on the same model structure used for the RT analysis that is presented in (1). The pattern of results was also highly comparable. Again, we observed significant switch costs, χ^2^(1) = 28.90, *p* < .001, and valency effects, χ^2^(1) = 7.24, *p* = .007. *N* − 1 univalent trials were significantly more error prone than post-bivalent trials, as evidenced by the main effect of *N* − 1 Valency, χ^2^(1) = 13.68, *p* < .001. Importantly, the interaction between Valency and *N* − 1 Valency reached again significance, χ^2^(1) = 5.56, *p* = .018. This effect was further characterised by a three-way interaction involving all factors, χ^2^(1) = 6.04, *p* = .014. We decomposed this interaction via Holm-corrected planned contrasts. While in switch trials, valency effects were not present following neither univalent (1.3%), *z* = 1.95, *p* = .153, nor bivalent trials (1.3%), *z* = 1.43, *p* < .001, in repetition trials, the valency effects found following univalent trials (3.1%), *z* = 4.68, *p* < .001, were completely absent after bivalent trials (−0.7%), *z* = −0.47, *p* = .635.

### Discussion

In Experiment 1, we aimed at demonstrating that task-conflict control can be upregulated on a trial-to-trial basis, similar to what has been observed with response-conflict control. In line with our expectations, we observed a sequential modulation of the valency effect (VSE), conceptually similar to the sequential modulation of the congruency effect (CSE) known from the response-conflict literature. Also, we assessed whether such adjustments were task-specific, again in parallel with the response-conflict literature. We observed a VSE in task-repetition trials, but not in task-switch trials, similar to the CSE which is known to occur in task repetitions, but not in task switches (Kiesel et al., 2006).

Notably, our experimental design allowed us to keep under control some important confounds. First of all, associative phenomena, known to threaten the validity of the CSE (Hommel, 2004; Mayr et al., 2003), were controlled for by using different stimuli throughout the experiment. Second, by excluding post-incongruent trials from the analyses, we excluded confounds between CSE and VSE. Finally, a closer look at our data pattern revealed that the observed reduction of the valency effect after bivalent trials was mostly driven by reduced task conflict following bivalent trials (i.e., better performance in *N* − 1 bivalent-bivalent trials compared with *N* − 1 univalent-bivalent trials), rather than by increased task conflict in univalent trials following bivalent trials (i.e., worse performance in *N* − 1 bivalent-univalent trials compared with *N* − 1 univalent-univalent trials). This data pattern indicates that our results were not driven by the so-called bivalency effect (Meier et al., 2009; Woodward et al., 2003), which would have not necessarily implied cognitive control upregulation.

## Experiment 2

The existence of the VSE as observed in Experiment 1 substantiates our methodological concerns regarding the previous reports of the PVE. Indeed, previous Stroop studies investigating this effect did not control for local modulations in task control observed in Experiment 1, and hence the observed pattern of results does not necessarily indicate the use of proactive control (which is the usual interpretation of the PVE), but could also arise from reactive control mechanisms (which is the usual interpretation of the VSE). Therefore, in Experiment 2, we sought to replicate the PVE controlling for the VSE. If we are successful in replicating such global effect while excluding local control adjustments, not only we will be able to advocate more convincingly for the use of proactive control when dealing with stimulus-based task conflict, in line with the Stroop literature, but also we would be able to demonstrate that global control adjustments also occur in the task-switching paradigm where the task-relevant dimension can switch in any trial.

### Methods

#### Participants

Sixty new participants took part in Experiment 2. The experiment was run online using Gorilla (Anwyl-Irvine et al., 2020).

#### Stimuli

The employed stimuli were built in the same way as described for Experiment 1. Due to the different composition of the blocks however, a different number of univalent and bivalent stimuli were chosen. For each of these two categories, 96 stimuli were chosen randomly among all the possible combinations. In particular, for each combination of task (Colour, Character) and majority level (Feature A, Feature B), we chose 24 (instead of 16) univalent stimuli. For bivalent stimuli, 12 were randomly chosen among the valid combinations for task (Colour, Character), majority level (Feature A, Feature B) and minority level (Feature A, Feature B). In Experiment 2, the use of different stimuli on each trial was of great importance, as it allowed us to exclude possible contingency learning confounds that could have arisen from the higher rate of presentation of individual stimuli (Schmidt, 2013).

#### Trial procedure

The trial procedure was identical to that of Experiment 1.

#### Experimental procedure

The experimental procedure was similar to that of Experiment 1 with a few noticeable exceptions. First of all, the experiment was split into two sessions separated by at least 1 day (maximum of 1 week). The sessions differed in respect of the proportion of univalent/bivalent trials used in each block. In one session, 75% of the trials employed univalent stimuli, with the remaining being bivalent stimuli. The other session presented 75% bivalent stimuli and 25% univalent stimuli. The order of the session was counterbalanced between participants.

Each session consisted of 1,024 trials divided in eight blocks of 128 trials each. Compared with Experiment 1 therefore, one block was removed for each session. This was done to ensure an equal number of majority-bivalent and majority-univalent blocks. While in Experiment 1 we used the same stimuli in each block, this was not possible in Experiment 2 due to the proportion of valency manipulation. As such, in each majority-univalent block, all univalent stimuli were used (*N* = 96), whereas a subset of 32 randomly selected bivalent trials was used, balanced for task and congruency. In majority-bivalent blocks, all bivalent stimuli were used, and a subset of 32 randomly selected univalent trials was chosen balancing for task and majority feature.

The sequence of task and stimuli was determined pseudorandomly as specified for Experiment 1, except that in Experiment 2 we did not balance for *N* − 1 Congruency. A graphical representation of the number of trials in each condition is given in Figure A2 of the Online Supplementary Material 1. Despite incongruent trials were again removed from the analyses, we still included them in our experimental design for the same reasons explained in Experiment 1, namely that not including incongruent trials would result in a lack of need to actually select a specific task.

#### Data trimming and statistical design

Data trimming was implemented following the procedures specified in the pre-registration protocol: https://aspredicted.org/c47rk.pdf.

Data trimming was similar to Experiment 1 with only one exception. Instead of removing *N* − 1 bivalent-incongruent trials, we removed all *N* − 1 bivalent trials to deconfound the PVE effect from the VSE shown in Experiment 1. Following our exclusion criteria, 31.3% of the trials were retained for the included participants. As for Experiment 1, most of the trials were forcefully excluded by design (60%; see Figure A2, in the Online Supplementary Material 1). We removed data sets from four participants from analysis due to excessive inaccuracy (i.e., >33% errors) and three participants due to insufficient number of trials (i.e., <15 trials in any condition of our design).

Statistical analyses diverged again from the pre-registration since, following the suggestion of an anonymous reviewer, we opted for modelling RTs with LMM, and error data with logistic regression. For both analyses, we used a 2 × 2 × 2 design with fixed effects Task Transition (repetition, switch), Valency (univalent, bivalent), and Block Type (majority univalent, majority bivalent). The random effect structure was again determined with a backward elimination procedure as illustrated for Experiment 1. Statistical inference was also performed in the same fashion. The originally planned analyses of variance (ANOVAs) can be found in the Online Supplementary Material 2. Furthermore, the interested reader can also find analyses including congruency as factor.

As specified in the pre-registration protocol, we predicted to find decreased valency effects in majority-bivalent blocks compared with majority-univalent blocks, indicating the presence of a proactive control mechanism reducing task conflict. In addition, we explored whether this effect differed for repetition and switch trials.

### Results

#### Reaction times

**Figure 3.**
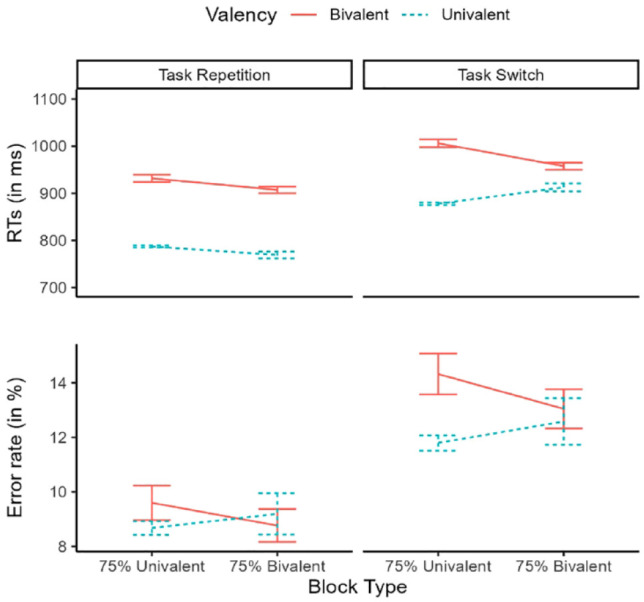
Experiment 2: descriptive statistics of the sample. Reaction times (upper panel) and error rates (lower panel) are plotted as a function of Valency (univalent, bivalent), Block Type (75% univalent, 75% bivalent), and Task Transition (repetition, switch). Error bars represent standard error of the mean.

Summary statistics of the sample’s RTs are plotted in the upper panel of Figure 3. The backward selection procedure held a random structure including by-subject random intercepts and correlated Task Transition, Valency, Block Type, and Valency × Task Transition random slopes. The residuals of this model however were strongly skewed. We therefore log-transformed the RTs and performed the same steps again. The same model was selected as before:

(2) logRT ~ Valency * Block Type * Task Transition + (Valency + Block Type + Task Transition + Valency:Task Transition | PP)

We found robust main effects of Task Transition, *F*(1, 62) = 183.21, *p* < .001, and Valency, *F*(1, 54) = 143.80, *p* < .001, indicating the emergence of switch costs and valency effects, respectively. These factors interacted significantly, *F*(1, 75) = 29.12, *p* < .001, indicating larger valency effects in task repetitions compared with switches. Critically, we found an interaction between Valency and Block Type, *F*(1, 33,831) = 50.63, *p* < .001, which was further modulated by Task Transition, *F*(1, 33,827) = 24.14, *p* < .001. To decompose this interaction, we proceeded with the planned contrasts. In repetition trials, valency effects were similar in majority-univalent (151 ms), *z* = 13.85, *p* < .001, and majority-bivalent blocks (141 ms), *z* = 10.78, *p* < .001. However, in switch trials, valency effects were still large in majority-univalent blocks (135 ms), *z* = 11.57, *p* < .001, but were much reduced in majority-bivalent blocks (47 ms), *z* = 3.47, *p* = .001.

#### Error rates

Summary statistics can be found in the lower panel of Figure 3. The model identified by our model selection procedure included correlated by-participants random intercepts and random slopes for all main effects. In formula,

(3) Error ~ Valency * Block Type * Task Transition + (Valency + Block Type + Task Transition | PP)

This model revealed again significant switch costs, χ^2^(1) = 42.49, *p* < .001, and valency effects, χ^2^(1) = 4.84, *p* = .028. The interaction between Valency and Block Type was the only other effect to approach significance, χ^2^(1) = 3.12, *p* = .077, whereas the three-way interaction involving all factor was far from significance, χ^2^(1) = 0.01. We thus proceeded to decompose the two-way interaction via Holm-corrected planned contrasts. Valency effects were found in majority-univalent blocks (1.7%), *z* = 3.27, *p* = .002, whereas they disappeared in majority-bivalent blocks (0.0%), *z* = 0.43, *p* = .435.

### Discussion

In Experiment 2, we demonstrated that the PVE reported in the Stroop literature also occurs in a task-switching paradigm, while excluding important confounds. First, we ruled out the possibility that trial-to-trial sequential effects produced the effect without invoking proactive control mechanisms. Second, using different stimuli in each trial, we were also able to control for contingency learning (Schmidt, 2013), which also confounded the results in previous PVE studies.

Although we did not have clear predictions of whether the PVE would be task specific (i.e., limited to repetition trials), or task general (i.e., observed in both repetition and switch trials), the observation that the PVE was limited to switch trials is rather surprising. One possibility is that in majority-univalent blocks, participants encode the task cue only shallowly and rely mostly on the target to provide a response (cf. Schneider, 2015). If cue-based task preparation is not really accomplished in majority-univalent blocks, we could expect task interference to be stronger in those trials where a new stimulus dimension is relevant, i.e., in task switches. In this sense, the observed interaction of the PVE with Task Transition could be conceptualised as an interaction between stimulus-based task conflict and carryover interference from the previous task-set. We discuss this account further in the general discussion.

## General discussion

Cognitive control deployment is thought to be flexibly regulated depending on the environmental circumstances. When the cognitive system deals with response conflict (measured via the congruency effect), previous literature demonstrated that cognitive control can act both globally and locally: at a global level, the congruency effect is reduced when response conflict is likely to occur in a block; at a local level, the congruency effect is reduced immediately after a response-conflict trial. In this study, we investigated whether such local and global control adjustments also occur when the cognitive system deals with stimulus-based task conflict. We measured stimulus-based task conflict via valency effects, which is the performance difference between bivalent congruent trials and univalent trials. We demonstrated that this type of task conflict is reduced after task-conflict trials (Experiment 1), and when task conflict is likely to occur in a block (Experiment 2), indicating local and global control adjustments, respectively.

To our knowledge, this is the first report of local (trial-by-trial) control adjustments to stimulus-based task conflict, as previous literature characterising the mechanisms of stimulus-based task-conflict control has mostly focused on global (blockwise) adjustments of control. For example, a computational model of the Stroop task has recently been developed, extending the well-known Botvinick model for conflict resolution (Botvinick et al., 2001), to include a task-conflict resolution mechanism that depends on the likelihood of encountering bivalent trials (Kalanthroff et al., 2018). Moreover, most of the studies investigating the adjustments to stimulus-based task conflict have manipulated the proportion of bivalent trials blockwise to engage/relax proactive control (Entel et al., 2015; Entel & Tzelgov, 2018; Goldfarb & Henik, 2007; Kinoshita et al., 2018). Therefore, our finding of a significant VSE advances the understanding of task-conflict control by revealing that reactive control mechanisms can also be recruited to aid task selection. In this sense, a parallel can be drawn with response conflict, which is known to decrease following incongruent trials (Gratton et al., 1992) producing the CSE. Also in line with the response-conflict literature, we found that the VSE was confined to task-repetition trials (cf. Kiesel et al., 2006). This finding supports the idea that cognitive control acts in a domain-specific manner (Braem et al., 2014; Egner, 2007, 2017) operating on the source of conflict, rather than in a domain-general fashion.

However, one important difference between response and task conflict should be noted. When measuring response conflict via the congruency effect, the task-irrelevant feature is always present in both congruent and incongruent trials, activating the relevant and irrelevant response, respectively. For this reason, inhibition of the irrelevant task feature is not advantageous if participants are presented with a congruent trial, as evidenced by the typical slowing in *N* − 1 incongruent-congruent trials compared with *N* − 1 congruent-congruent trials. When measuring task conflict instead, the task-irrelevant feature is only present in bivalent trials, but not in univalent trials. For this reason, inhibition of the task-irrelevant feature during bivalent trials should not influence performance in the subsequent univalent trial. In support of this prediction, we found that performance in *N* − 1 bivalent-univalent trials was not significantly slower than *N* − 1 univalent-univalent trials in both experiments. This finding is also relevant because it indicates that the observed effects are not due to a shift towards a more cautious response style following infrequent trial types (Woodward et al., 2003), or to episodic context binding (Meier et al., 2009; Rey-Mermet & Meier, 2012), as suggested by previous literature on the bivalency effect. Nonetheless, it is to be noticed that in both experiments, a numerical slowing in *N* − 1 bivalent-univalent trials was observed, so that we cannot completely exclude a contribution of these phenomena.

### Proactive task-conflict control

The finding of a large VSE substantiated our concerns about a possible confound in previous Stroop studies investigating the PVE (e.g., Goldfarb & Henik, 2007). In particular, the data pattern observed in those studies could be either due to proactive control (as indicated by the PVE) or to reactive control (as indicated by the VSE, which was not measured in those earlier studies). In this study, we controlled for this possible confound by excluding all post-bivalent trials from analyses when computing the PVE in Experiment 2. Another methodological advantage of the paradigm employed here was that we used a large stimulus set, which allowed us to exclude contingency learning phenomena (Schmidt, 2013). The use of small stimulus sets is problematic because manipulating the proportion of congruent/incongruent or bivalent/univalent trials necessarily leads to a higher rate of presentation of the individual stimuli belonging to the over-represented category. For example, the word RED printed in red will occur much more frequently than RED printed in black in majority-bivalent blocks. Under such circumstances, the observed PVE may actually be due to facilitation of processing most familiar items, rather than cognitive control upregulation. Controlling for these two important confounds, we found that task conflict was still reduced in majority-bivalent blocks, at least in switch trials, replicating and extending results from the Stroop literature.

The finding of PVE in task switching is intriguing because it suggests that participants are able to reduce task conflict proactively. In single-task studies, this is thought to be achieved by selective suppression of the task-irrelevant feature or enhancement of the task-relevant feature. In task switching however, such sustained attentional adjustments are not possible because the relevant and irrelevant features may change on any trial. Therefore, it is likely that proactive control is exerted only after the task cue for the upcoming task is presented, namely during the CSI. Possibly, in majority-bivalent blocks, participants tend to prepare for the upcoming bivalent stimulus by focusing attention on the task-relevant features (or by inhibiting the task-irrelevant features). On the contrary, in majority-univalent blocks, participants may choose not to prepare at all, and simply rely on the stimulus to select a response (cf. Schneider, 2015). In this latter scenario, the occurrence of bivalent stimuli in majority-univalent blocks would trigger greater task conflict than in majority-bivalent blocks.

This account may also explain why the PVE was limited to task-switch trials. Even in the absence of cue-based task preparation, if the task repeats, participants can still rely on the same task-set that was used in the previous trial, in line with the notion of carryover activation of the previous task (Allport et al., 1994). For this reason, task conflict in majority-univalent and majority-bivalent trials could be similar, and overall smaller than in switch trials. In other words, the observed pattern of results may arise from an interaction between stimulus-based task conflict and task-set inertia. Future studies may test this idea further, for instance by manipulating the time for advance task preparation (i.e., by manipulating the CSI).

An alternative source of the PVE, not related to cognitive control, may be the expectation about the upcoming stimulus type. Infrequent events are generally known to slow down behaviour in speeded reaction time tasks (Barcelò et al., 2006; Notebaert et al., 2009). For example, the slowing observed following errors has been interpreted as a re-orienting response to a surprising event, as post-error slowing is eliminated when errors are frequent (Notebaert et al., 2009). Similarly, as mentioned above, presenting infrequent bivalent stimuli slows down performance in subsequent bivalent trials, which has also been interpreted in terms of a shift towards more cautious responding (Woodward et al., 2003). In our case, it is possible that participants expected to encounter bivalent stimulus in majority-bivalent blocks, whereas they expected univalent stimuli in majority-univalent blocks, slowing down whenever this expectation was violated. This pattern may be particularly strong in switch trials because participants may exert particularly strong proactive control on task conflict in those trials, as discussed above. Future studies may consider the issue of surprise evoked by infrequent stimuli further, for instance by using an equal proportion of univalent/bivalent trials in each block, and manipulating this proportion task-wise (e.g., the majority of trials in the colour task could be congruent, whereas the majority of trials in the character task could be incongruent). We believe this issue to be of great importance, as surprise-related slowing may be invoked for any proportion-of-conflict effect (e.g., PCE, PVE, proportion-switch effect).

## Conclusion

In this study, we aimed at investigating local and global control mechanisms involved in dealing with stimulus-based task conflict. For the first time, we showed that task-conflict control is regulated on a trial-by-trial basis depending on recent task-conflict history, as indicated by the VSE. In addition, such local adjustments of task-conflict control were found to be task-specific, drawing a strong parallel with the control of response conflict. Furthermore, based on previous Stroop studies indicating that participants proactively prepare for task conflict, we investigated whether the same mechanisms are also recruited in task switching where task conflict is brought about by different stimulus features. Controlling for the VSE and contingency learning phenomena, we found that stimulus-based task conflict was indeed reduced in majority-bivalent blocks as compared with majority-univalent blocks, although this was only true for task-switch trials. Although the precise mechanisms producing such PVE still demand further research, these findings indicate a strong parallel between task-conflict control and response-conflict control.

## Supplemental Material

sj-docx-1-qjp-10.1177_17470218231200442 – Supplemental material for Local and global control adjustments to stimulus-based task conflict in task switching Supplemental material, sj-docx-1-qjp-10.1177_17470218231200442 for Local and global control adjustments to stimulus-based task conflict in task switching by Luca Moretti, Iring Koch and Stefanie Schuch in Quarterly Journal of Experimental Psychology

## Notes

1. This computation was made having in mind an analysis of variance (ANOVA) design, as we initially planned to report the results of these analyses in the main text, as pre-registered. As specified below however, we followed the suggestions of an anonymous reviewer to compute and report linear mixed models and generalised linear mixed models for RT and error rates analyses, respectively.

2. We originally planned and computed ANOVAs for analysing both RTs and error rates. The results were qualitatively the same as those found with the linear mixed model (LMM) and generalised linear mixed model (GLMM). We report the originally planned ANOVAs in the Online Supplementary Materials.
