# Supplementary material for: Local and global control adjustments to stimulus-based task conflict in task switching
Source: Q J Exp Psychol (Hove). 2023 Sep 28;78(5):963–77. doi: 10.1177/17470218231200442 (PMC11992644; doi:10.1177/17470218231200442)
Supplement: sj-docx-1-qjp-10.1177_17470218231200442 – Supplemental material for Local and global control adjustments to stimulus-based task conflict in task switching [file sj-docx-1-qjp-10.1177_17470218231200442.docx]

Supplementary Material for:

**Local and global control adjustments to stimulus-based task conflict in task switching.**

Luca Moretti^1^, Iring Koch^1^, & Stefanie Schuch^1^

^1^Institute of Psychology, RWTH Aachen University, Aachen, Germany

Author Note

Part of this research was supported by grants within the Priority Program (SPP 1772) from the German Research Foundation (Deutsche Forschungsgemeinschaft, DFG); Grant No. KO 2045/19-2 awarded to Iring Koch; Grant No. SCHU 3046/1-2 awarded to Stefanie Schuch.

Pre-registration protocols are available at: <https://aspredicted.org/tx3jh.pdf> and <https://aspredicted.org/c47rk.pdf>. Raw data and analyses scripts are available at: <https://osf.io/9m4he/>.

Correspondence concerning this article should be addressed to Luca Moretti, Institute of Psychology, RWTH Aachen University, Jaegerstrasse 17/19, 52066 Aachen, Germany. Email: [Luca.Moretti@psych.rwth-aachen.de](mailto:Luca.Moretti@psych.rwth-aachen.de)

**Supplementary Material 1**

In both of our experiments many trials were discarded to avoid possible confounds with response-conflict effects (i.e. incongruent trials), the congruency sequence effect (i.e. post-incongruent trials) or, in Experiment 2, with trial-by-trial modulation of task-conflict control (i.e. post-bivalent trials). As a result, the interested reader may find hard to derive the final number of trials that were used in each experimental condition. In Figures A1 and A2 we provide a visualization of this important factor, together with a scheme that helps clarifying how this trial number was achieved, and which factors we controlled for when creating our trial sequences.

**Figure A1**

*Experiment 1: Number of trials in each experimental condition.*


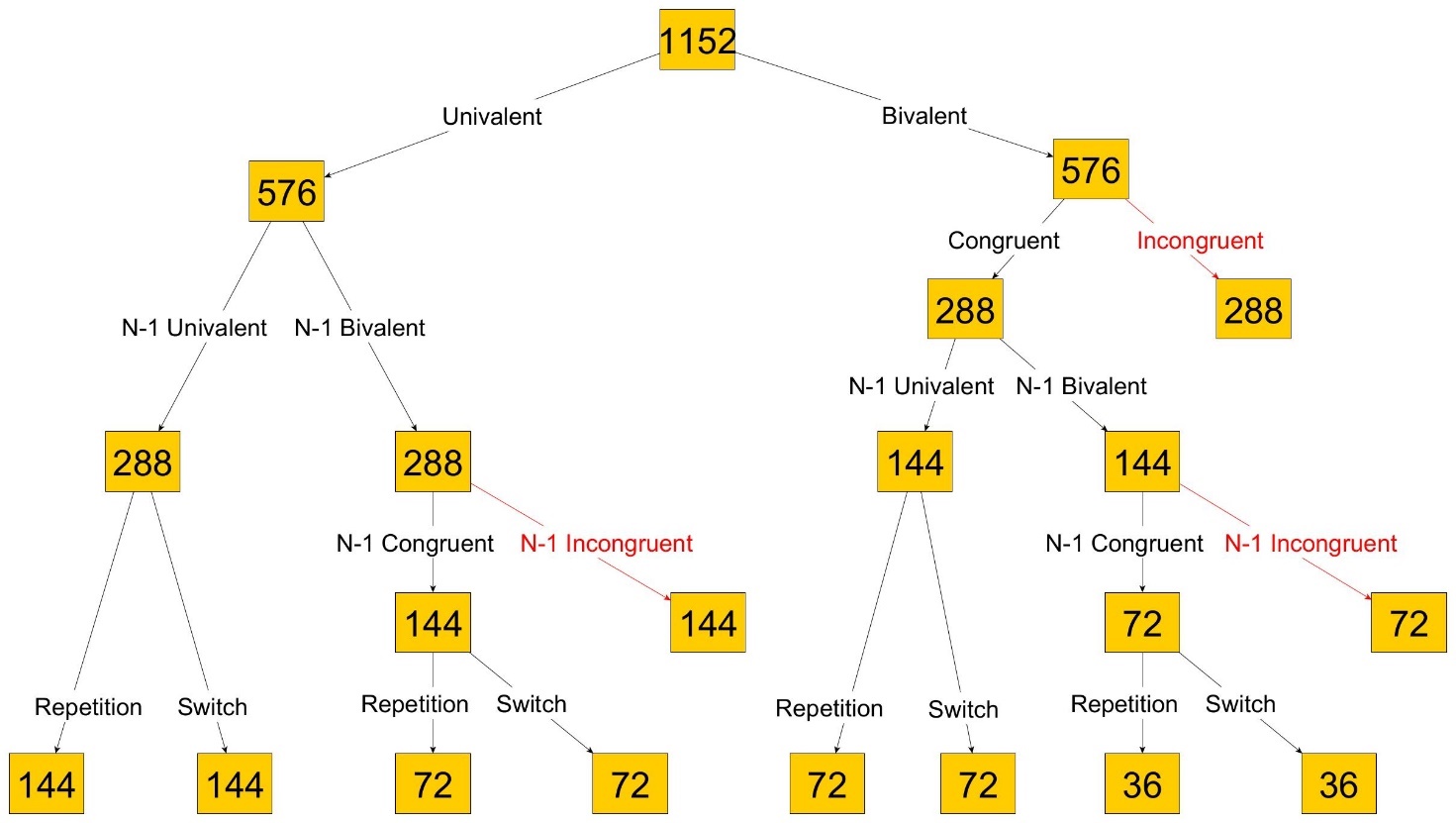


*Note:* Numbers inside the squares represent the number of trials in each condition. At the top of the tree are represented the number of trials in the whole experiment. These were then divided equally for each level of valency, congruency, N-1 congruency, N-1 valency and task transition. Branches in red represent conditions that were excluded from analysis. For example, although incongruent trials were also equally distributed between repetition and switch trials, this is not shown in the figure as incongruent trials were excluded from analysis a-priori. In this way, the bottom layer represents the number of trials included in each cell of our ANOVA design.

**Figure A2**

*Experiment 2: Number of trials in each experimental condition.*


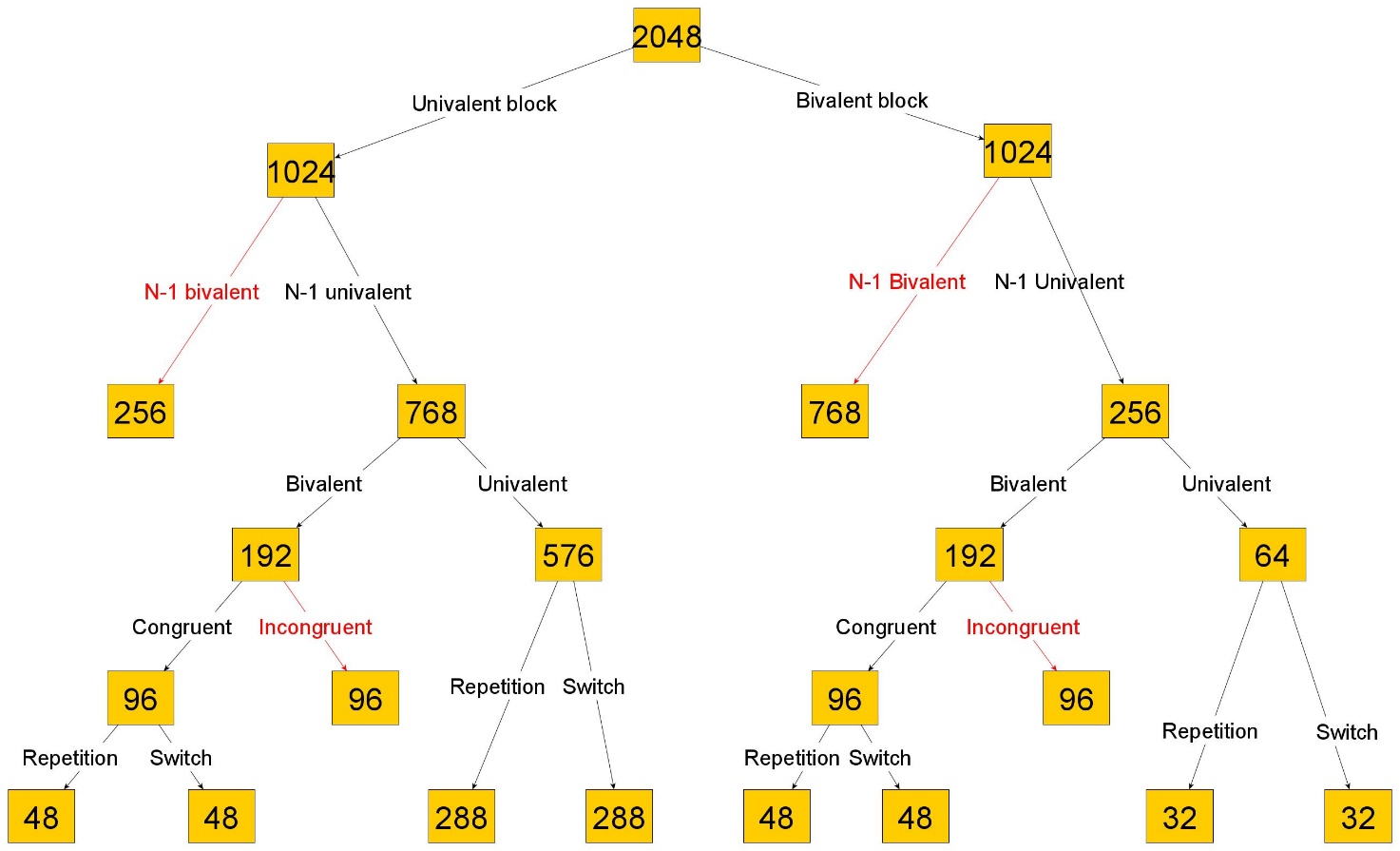


*Note:* Numbers inside the squares represent the number of trials in each condition. At the top of the tree are represented the number of trials in the whole experiment. These were then divided equally for each level of congruency, N-1 congruency, N-1 valency, and task transition. The proportion of trials allocated to each level of valency depended on the block type. Branches in red represent conditions that were excluded from analysis. For example, although incongruent trials were also equally distributed between repetition and switch trials, this is not shown in the figure as incongruent trials were excluded from analysis a-priori. In this way, the bottom layer represents the number of trials included in each cell of our ANOVA design.

**Supplementary Material 2**

**Originally planned ANOVA analyses**

In the pre-registration protocols of Experiment 1 and 2, we planned to run analyses for both RTs and error rates using ANOVAs. As one anonymous reviewer noticed howerver, ANOVA designs are not apt to the analyses of error rates (Jaeger, 2008). As such, we decided to analyse the data in the main text using linear-mixed models for RTs and generalized linear mixed models for errors. The ANOVA results originally present in the text have therefore been moved to this appendix in compliance with our pre-registered plans.

**Experiment 1**

Following data trimming as described in the main text, we conducted separate ANOVAs on RTs and square root-arcsine transformed error rates. Independent variables were Valency (Bivalent, Univalent), N-1 Valency (Bivalent, Univalent), and Task Transition (Repetition, Switch). We expected to find a significant interaction between Valency and N-1 Valency, which could possibly be limited to repetition trials.

**Results**

***Reaction times.*** Summary statistics of the sample’s RTs are plotted in the upper panel of Figure 1 of the main text. In the RT analysis we observed main effects of Task Transition, *F*(1, 52) = 210.18, *p* < .001, $\eta_{p}^{2}$ = .80, $\eta_{G}^{2}$ = .09, and Valency, *F*(1, 52) = 123.11, *p* < .001, $\eta_{p}^{2}$ = .70, $\eta_{G}^{2}$ = .05 indicating the presence of switch costs and of the valency effect respectively. Also, post-bivalent trials were overall significantly slower than post-univalent trials *F*(1, 52) = 12.16, *p* = .001, $\eta_{p}^{2}$ = .19, $\eta_{G}^{2}$ < .01. Critically for our hypothesis, Valency and N-1 Valency interacted significantly, *F*(1, 52) = 41.00, *p* < .001, $\eta_{p}^{2}$ = .44, $\eta_{G}^{2}$ = .01, indicating that the valency effect was reduced following bivalent trials. Finally, this effect was further characterized by a 3-way interaction involving all factors, *F*(1, 52) = 32.56, *p* < .001, $\eta_{p}^{2}$ = .39, $\eta_{G}^{2}$ < .01. In order to decompose this interaction, we performed separate 2-way ANOVAs for repetition and switch trials separately. In repetition trials we found a large interaction between Valency and N-1 Valency *F*(1, 52) = 94.70, p < .001, $\eta_{p}^{2}$ = .44, $\eta_{G}^{2}$ = .02, indicating that, while the valency effect was very robust following univalent trials (115 ms), *t*(52) = 14.63, *p* < .001 , *d_z_* = 2.01, it was reduced following bivalent trials (27 ms), *t*(52) = 3.76, *p* < .001, *d_z_* = 0.52. On the other hand, such an interaction was not significant in task switches *F*(1, 52) < 1. Valency effects were found both in N-1 univalent trials (74 ms), *t*(52) = 8.52, *p* < .001, *d_z_* = 1.17 and in N-1 bivalent trials (67 ms), *t*(52) = 5.86, *p* < .001, *d_z_* = 0.81.

***Error rates.*** The error rates ANOVA revealed main effects of Task Transition, *F*(1, 52) = 35.53, *p* < .001, $\eta_{p}^{2}$ = .41, $\eta_{G}^{2}$ = .05, indicating the presence of switch costs, and of N-1 Valency, *F*(1, 52) = 24.99, *p* < .001, $\eta_{p}^{2}$ = .32, $\eta_{G}^{2}$ = .02, indicating higher accuracy following bivalent trials. The main effect of Valency did not reach significance *F*(1, 52) = 2.52, *p* = .118, $\eta_{p}^{2}$ = .05, $\eta_{G}^{2}$ < .01. Critically, Valency interacted significantly with N-1 Valency, *F*(1, 52) = 9.65, *p* = .003, $\eta_{p}^{2}$ = .16, $\eta_{G}^{2}$ < .01, due to larger valency effects following bivalent trials. As in the RT analysis, this effect was further characterized by a 3-way interaction involving all factors, *F*(1, 52) = 4.42, *p* = .040, $\eta_{p}^{2}$ = .08, $\eta_{G}^{2}$ < .01. In order to decompose this effect, we run separate 2-way ANOVAs on each level of Task Transition separately. In repetition trials we found a significant 2-way interaction between Valency and N-1 Valency, *F*(1, 52) = 13.99, *p* < .001, $\eta_{p}^{2}$ = .21, $\eta_{G}^{2}$ = .03. While the valency effect was present following univalent trials (3.1%), *t*(52) = 4.45, *p* < .001, *d_z_* = 0.61, it was even numerically reversed following bivalent trials (-0.7%), *t*(52) < -1. On the other hand, the same 2-way interaction did not reach significance in switch trials, *F*(1, 52) < 1. Here, valency effects were neither significant following univalent (1.3%), *t*(52) = 1.53, *d_z_* = 0.21, nor bivalent trials (1.3%), *t*(52) < 1.

**Experiment 2**

Following data trimming we performed separate 2 x 2 x 2 ANOVAs on RTs and square-root arcsine transformed error rates, with factors Task Transition (Repetition, Switch), Valency (Univalent, Bivalent) and Block Type (Majority Univalent, Majority Bivalent). Higher order interactions were decomposed by performing separate tests on each level of a specific factor of interest (e.g. 2-way ANOVAs for repetition and switch trials separately).

**Results**

***Reaction times.*** Summary statistics of the sample’s RTs are plotted in the upper panel of Figure 2 of the main text. The 3-way ANOVA revealed both the presence of overall switch cost, *F*(1, 52) = 200.54, *p* < .001, $\eta_{p}^{2}$ = .79, $\eta_{G}^{2}$ = .06, and valency effect, *F*(1, 52) =169.69, *p* < .001, $\eta_{p}^{2}$ = .77, $\eta_{G}^{2}$ = .10. These two factors did interact significantly, *F*(1, 52) = 32.23, *p* < .001, $\eta_{p}^{2}$ = .38, $\eta_{G}^{2}$ = .01, indicating larger valency effects in task repetitions compared to switches. Critically, Valency also interacted significantly with Block Type, due to a reduction of the valency effect in Majority Bivalent blocks. *F*(1, 52) = 24.53, *p* < .001, $\eta_{p}^{2}$ = .32 $\eta_{G}^{2}$ < .01. This effect was further characterized by a 3-way interaction involving all factors, *F*(1, 52) = 20.14, *p* < .001, $\eta_{p}^{2}$ = .28, $\eta_{G}^{2}$ < .01. In order to decompose this interaction, we run separate 2-ways ANOVAs for task switch and task repetition trials separately. In task switches, we found a strong interaction between Valency and Block Type, *F*(1, 52) = 48.25, *p* < .001, $\eta_{p}^{2}$ = .48, $\eta_{G}^{2}$ = .01. The valency effect was present both in majority-univalent, (134 ms), *t*(52) = 10.81, *p* < .001, *d_z_* = 1.49, and majority-bivalent blocks (48 ms), *t*(52) = 3.48, *p* = .001, *d_z_* = 0.48, but were reduced in the latter. On the other hand, no such interaction emerged for repetition trials, *F* < 1. Strong valency effect emerged both in majority-univalent blocks (152 ms), *t*(52) = 12.04, *p* < .001, *d_z_* = 1.65, and in majority-bivalent blocks (141 ms), *t*(52) = 14.38, *p* < .001, *d_z_* = 1.98.

***Error rates.*** Summary statistics can be found in the lower panel of Figure 2 of the main text. The only significant effect concerning error rates was the main effect of Task Transition, indicating the presence of swith cost, *F*(1, 52) = 68.54, *p* < .001, $\eta_{p}^{2}$ = .57, $\eta_{G}^{2}$ = .06. No other effect reached or approached significance, *p*s > .131.

**Congruency effect analyses**

As mentioned in the manuscript, incongruent trials were included in the experimental design for both experiments. This was done so to avoid that task selection was made unnecessary. In other words, although congruency was manipulated, this was done for purely methodological reasons. Nonetheless, congruency effects may also be modulated by N-1 valency and the proportion of bivalent trials in a block. Although we do not have specific predictions in this regard, here we report these exploratory analyses.

**Experiment 1**

Data trimming was similar to that described in the main text, except that now univalent trials were excluded from analyses, whereas incongruent trials were not. The factor Valency was now substituted with Congruency, so that the statistical design now included the factors Task Transition (repetition, switch), Congruency (congruent, incongruent) and N-1 Valency (univalent, bivalent). Participants’ data were excluded from analyses if there were less than 15 trials in any cell of this design (4 participnats).

**Results**

***Reaction times.*** Descriptive statistics are depicted in Figure S1. The model-selection procedure held a model whose residuals’ distribution violated the normality assumption. As such, the data were log-transformed and the same procedure was again applied. The final structure of our model was:

1. logRT ~ Congruency + N-1 Valency + Task Transition + N-1 Valency:Task Transition + (Congruency + Task Transition | PP)

Only the main effects of N-1 Valency *F*(1, 15,205) = 79.59, *p* < .001 and Task Transition *F*(1, 54) = 227.04, *p* < .001 reached significance, indicating that performance was overall faster following bivalent trials, and the presence of switchcsots. The main effect of Congruency instead, did not reach significance, *F*(1, 47) = 2.62, *p* = .112.

***Error rates.*** The backward procedure resulted in selecting the model:

1. logRT ~ Congruency + N-1 Valency + Task Transition + Congruency:Task Transition + (Congruency + Task Transition | PP)

Testing the model’s parameters we found all main effects to be significant. In addition to the main effects of Task Transition, χ^2^(1) = 37.15, *p* < .001 and N-1 Valency, χ^2^(1) = 20.68 which were in the same direction as the RT analyses, we also found a significant congruency effect χ^2^(1) = 37.03, *p* < .001. This factor interacted significantly with Task Transition χ^2^(1) = 10.49, *p* = .001, revealing stronger congruency effects for switch compared to repetition trials.

**Discussion**

In Experiment 1 we observed significant congruency effects limited to the error rates analyses. Although the lack of a main effect of congruency in RTs may seem odd, it is actually common in task switching research to observe larger congruency effects in the error rates compared to RTs. In particular, the fact that our congruency effects were large in error rates (M_incong_ = 21.3%, M_univalent_ = 11.6%), but modest in RTs (M_incong_ = 932 ms, M_univalent_ = 925 ms), replicates previous studies using a similar deadline procedure as ours (Steinhauser & Hübner, 2006; 2008). With such a difficult task and stringent deadline procedures, it is possible that more variance across conditions would be captured by the error rates, rather than RTs (cf. Steinhauser & Hübner, 2006). However, this is a purely speculative interpretation, and empirical data would be needed manipulating response deadline. Also in line with previous studies, is the finding that congruency effects were larger in switch trials (Kiesel et al., 2010), possibly indicating an increased activaiton of the irrelevant S-R rules.

Finally, it is interesting to notice that N-1 Valency and Congruency did not interact in the erorr rates. As we do not provide a test for the null hypothesis (e.g., using the Bayes factor), these results should be interpreted with caution. However, from the lower panel Figure S1 it is apparent that the lack of a significant interaction derives from the fact that perfromance improved following bivalent trials irrespective of the stimulus’ congruency. This result aligns well with our expectations as stimulus-based task confict is present in both congruent and incongruent trials. It is thus reasonable to expect that higher task-conflict control would result in improved performance, not only incongruent, but also in incongruent trials. Moreover, the lack of interaction between N-1 Valency and Congruency may indicate that response conflict, as reflected by the congruency effect, was not affected by N-1 Valency, thus suggesting that task-conflict control does not transfer to response-conflict control.

**Figure S1**

*Experiment 1: descriptive statistics of the sample*
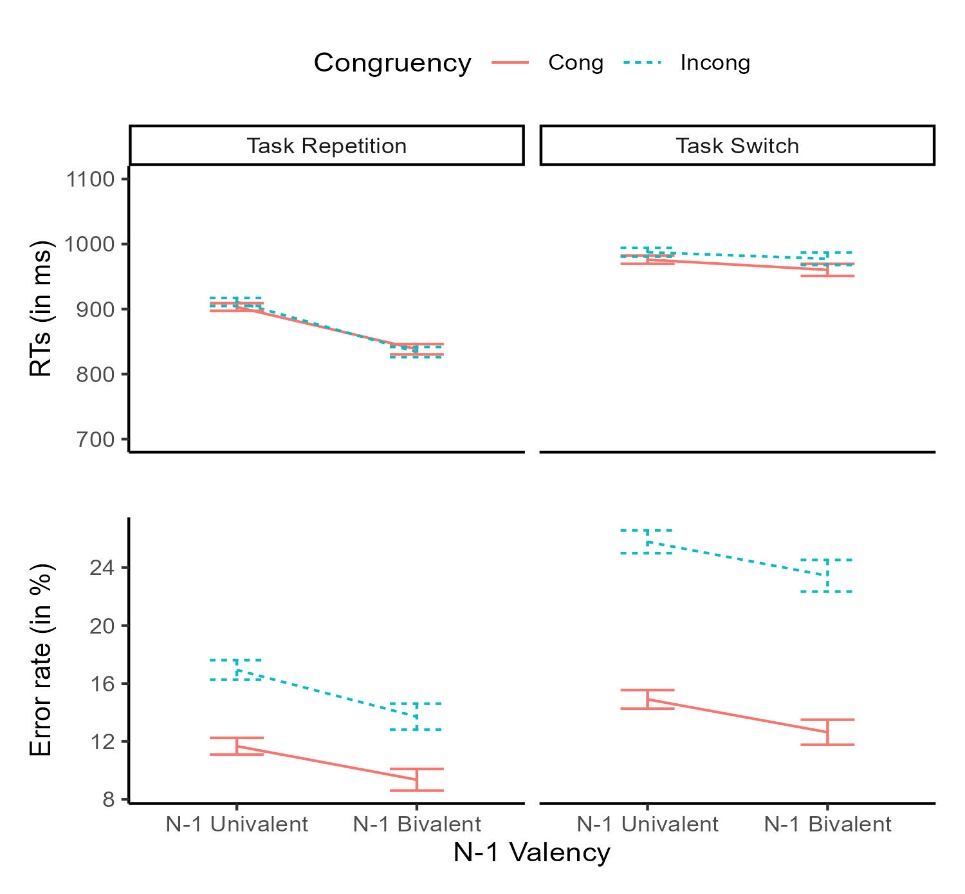


*Note:* Reaction times (upper panel) and error rates (lower panel) are plotted as a function of Congruency (congruent, incongruent), N-1 Valency (univalent, bivalent), and Task Transition (repetition, switch). Error bars represent standard error of the mean.

**Experiment 2**

In Experiment 2 we again replaced the factor Valency with Congruency, and run the same analyses as reported in the main text.

**Results**

***Reaction times.*** Descriptive statistics of Experiment 2 are depicted in Figure S2. Our model selection procedure resulted once again in the selection of a model whose residuals’ distribution violated normality. We thus run the same procedure on log-transformed RTs and selected the following model:

1. logRT ~ Task Transition * Block Type + (Task Transition * Block Type | PP)

As the Congruency factor was not present in this model, we do not report any further results.

***Error rates.*** In the error rates analyses, the final model structure was:

1. ER ~ Task Transition * Block Type * Congruency + (Block Type + Congruency | PP)

The main effect of Task Transition χ^2^(1) = 130.49, *p* < .001, and Congruency χ^2^(1) = 60.61, *p* < .001 indicated the presence of the switch cost and congruency effects, respectively. The interaction between these factors approached significance χ^2^(1) = 3.63, *p* = .056, indicating marginally larger congruency effects in switch trials than in repetition trials. Finally, we observed a main effect of Block Type, χ^2^(1) = 5.01, *p* = .025, indicating that performance was more error prone in 75% Univalent blocks.

**Discussion**

The results reported in the present analysis largely replicate those of Experiment 1. First, the overall congruency effect was limited to error rates, and absent in RTs (cf. Steinhauser & Hübner, 2006; 2008). Second, congruency effects were larger in switch trials compared to repetition trials (cf. Wendt & Kiesel, 2008). Third, there was no interaction between Block Type and Congruency, althogh there was a main effect of Block Type, indicating that overall less errors were committed in 75% bivalent blocks. However, it is hard to interpret these results due to a variety of reasons. First, as for Experiment 1, we do not provide a test of the null hypothesis for the interaction. Second, the pattern of results seems to be less clear, with performance improvements that are only apparent in incongruent switch trials. Third, in Experiment 2 we did not control for N-1 congruency, so that the observed results may be due to different proportions of congruency in the previous trial (i.e., CSE).

**Figure S2**

*Experiment 2: descriptive statistics of the sample*


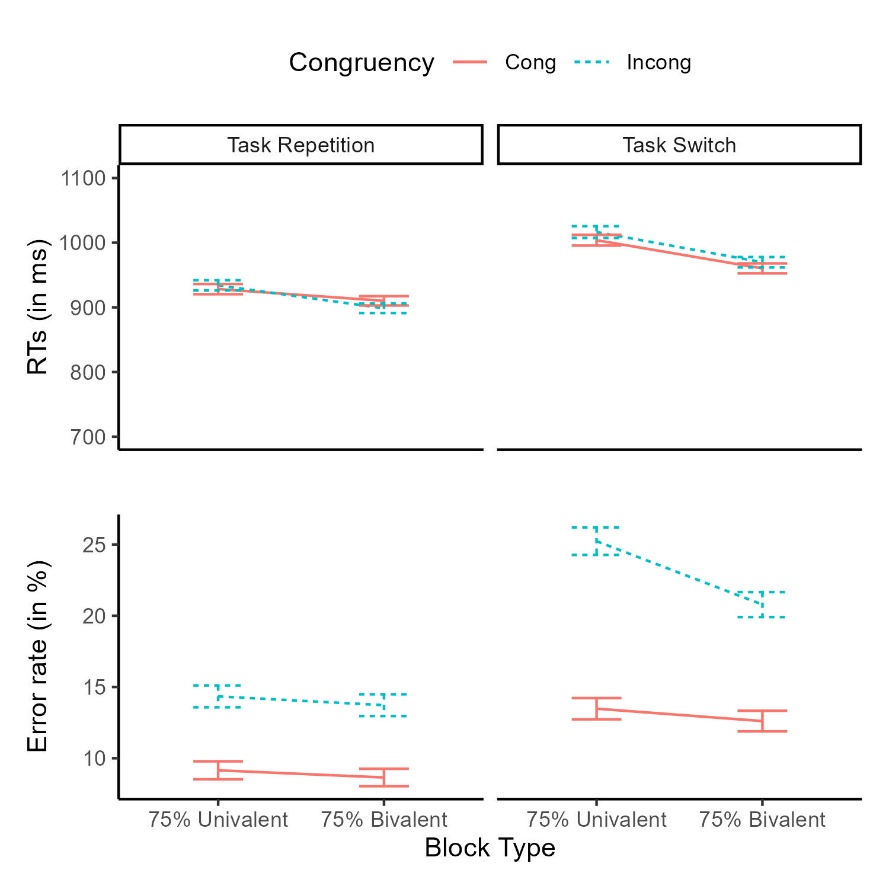


*Note:* Reaction times (upper panel) and error rates (lower panel) are plotted as a function of Congruency (Congruent, Incongruent), Block Type (75% Univalent, 75% Bivalent), and Task Transition (Repetition, Switch). Error bars represent the standard error of the mean.
